# Household Hints and Recipes

**Published:** 1874-10

**Authors:** 


### Household Hints & Recipes.

TO BLEACH SPONGES.—Soak them in very dilute muriatic acid ten or twelve hours, wash them in water and immerse them in a solution of sulphite of soda acidulated with muriatic acid. Wash them again, and repeat the process until they are sufficiently white.

---

CEMENT FOR AQUARIA.—An adhesive cement for aquaria may be made, according to Klein, by mixing equal parts of flowers of sulphur pulverized, salammoniac, and iron filings, with good linseed-oil varnish, and then adding enough of pure white lead to form a firm, easily worked mass.

---

PRESERVATION OF ESSENCE OF LEMON.—The addition of two ounces of water to each pound of essence of lemon insures its preservation, owing to the water producing coagulation and precipitation of the mucilaginous substances which cause resinification of the essence. Following this treatment the essence will preserve its odor for several years.—*The Pharmacist.*

---

MUSHROOM CATSUP.

| | |
|---|---|
| Mushroom juice | 10 gallons. |
| Black pepper, bruised | 14 ounces. |
| Allspice | 12 " |
| Ginger | 8 " |
| Cloves | 1 " |
| Salt | 5 pounds. |

Gently simmer in a covered tin boiler for one hour.

---

PROPORTION OF ALCOHOL IN VARIOUS COMMERCIAL BITTERS.—The State Assayer of Rhode Island has been making an examination of the so-called bitters found in the American market, and, of thirty-four samples, finds the amount of alcohol to run from 6.36 to 43.20 per cent

The following list contains an enumeration of those bitters which are best known in the markets:

| | |
|---|---|
| Hostetter's Stomach Bitters | 43.20 |
| Drake's Plantation Bitters | 30.24 |
| Rush's Bitters | 34.30 |
| Peruvian Bitters | 22.40 |
| Hoofland's German Bitters | 20.85 |
| Oxygenated Bitters | 19.20 |
| California Wine Bitters | 18.20 |
| Walker's Vinegar Bitters | 7.50 |

A Substitute for Milk or Cream.—Beat up the whole of a fresh egg in a basin, and then pour boiling tea over it gradually, to prevent its curdling. It is difficult from the taste to distinguish the composition from rich cream.

Corns.—Bathe the feet well in warm water, then with a sharp instrument pare off as much of the corn as can be done without pain or causing it to bleed, and dress once a day with the following salve:—

℞ Black oxide of copper.........gr. xv.
Lard.......................... oz. ss.
Mix.

To Remove Iron Rust From Clothing.—Make a mixture of two parts oxalic acid and one part citric acid, both finely powdered; sprinkle some of it on the stain previously moistened with water, and expose to the sunlight. When the rust has disappeared, wash the fabric thoroughly in cold water.

Salad Dressing.—This is excellent both for salad and for sliced tomatoes in summer. Take the yolk of one fresh egg and mix it with two table-spoonfuls of olive oil very slowly, add one and one half spoonfuls of mustard, three spoonfuls of salt, a little pepper, and last of all, two spoonfuls of vinegar. Beat the white of the egg to a stiff froth, and lightly stir in.

Soft Cheese.—Take milk just as it begins to turn sour; pour over it about one fourth its bulk of scalding water, beating the milk with a spoon at the same time to cause the whey to separate. Then strain off as much of the liquid as possible, finally washing the curd with clean water. Add a little salt, and you have a palatable and very nutritious article of food.

Crimson Marking Ink.—The following has been recommended:—

Powdered cochineal.
Cream of tartar, of each........2 ounces.
Carbonate of potassa..........1 ounce.
Alum,
Gum arabic, of each...........1 "
Boiling water, sufficient.

Boil the cochineal and the cream of tartar with 8 ounces of water, strain, add the carbonate of potassa and, lastly, the alum and the gum arabic. Should the color of the liquid partake too much of the scarlet shade, add enough carbonate of potassa to turn it to the crimson hue which you desire.

Hair Curling.—The following is said to be satisfactory:—

Carbonate of potassa........30 grains.
Ammonia...................1 drachm.
Glycerine..................2 drachms.
Alcohol....................12 "
Water......................18 ounces.

Mix, color and scent according to taste, and filter. The hair is moistened with it, and then loosely adjusted. The effect occurs as it dries.

Should this cosmetic cause your hair to curl too closely to suit your notions of propriety, or your idea of manly beauty, you will find in our back numbers an abundance of receipts for straightening curly hair.

Walnut Catsup.

Green walnut shells.........16 gallons.
Salt.........................5 pounds.

Mix, and beat together for a week. Press out the liquor, and to every gallon add:

Allspice, bruised............4 ounces.
Ginger, " ............3 "
Pepper, "
Cloves, " of each....2 "

Simmer for half an hour, and set aside in a closed vessel and in a cool place until sufficiently clear.

Shaving Fluid.

1. Take of

White hard soap (in shavings) .....¼lb.

Alcohol..........................1 pt.

Water............................¼ pt.

Perfume (at will).

Put them in a strong bottle, cork it close, set it in warm water for a short time, and occasionally agitate it briskly until solution is complete.

After repose, pour off the clean portion from the dregs into clean bottles for use, and at once closely cork them. If the solution be not sufficiently transparent, a little alcohol should be added to it before decantation.

2. Take of

White soft soap..................¼lb
Liquor of potassa..............2 fl. dr.
Alcohol........................1 pint.
Perfume (at will).

Proceed as before. The product of both is excellent.

By simply rubbing a few drops on the skin and applying the shaving-brush, previously slightly dipped in water, a good lather is produced.—*Druggists' Circular.*

Mock Venison of Corn Beef.—Cut the beef in thin slices, and freshen by soaking for three or four hours in tepid water. When sufficiently fresh, lay the slices on a gridiron, and heat through quickly. Make a gravy of drawn butter; add a little pepper, and the yolk of an egg chopped fine, and pour over the meat; or butter, pepper, and salt, like beefsteak. This will be found a savory dish when only salt meat can be procured, but it is better with fresh beef.

---

A Good Breakfast Dish.—The following is as good as it is easy to prepare: Take four eggs, three quarters of a pint of new milk, and a piece of butter the size of a walnut; salt (and pepper it if you like it) to suit the taste. Beat the eggs, add the milk and butter, and pour all together into a hot frying pan containing half a spoonful of lard or butter. Stir constantly for three or four minutes, when it will be ready for the table.

---

Indelible Red Ink.—Dr. Elsner states that an indelible red ink can be prepared by taking equal parts, by weight, of copperas and cinnabar, both in fine powder and sifted, and rubbing up with linseed oil with a muller, finally squeezing through a cloth. The thick paste can be employed for writing, or stamping woolen or cotton goods, and the color remains fast after the goods have been bleached. The reds usually employed are not fast colors, and do not resist the action of bleaching agents.

---

Pumpkin Preserves.—Cut a nice ripe pumpkin into pieces a third of an inch thick, paring them. Take equal weight in white sugar.—Allow the juice of one lemom to a pound of pumpkin. Let the pumpkin remain in a pan with the sugar and juice all night. In the morning put into a preserving kettle, cooking until perfectly clear. Be sure to skim well. Then add lemon peel cut in pieces small as marbles. Take out and strain the syrup through a jelly bag, and pour over the pumpkin.

---

Grease, Tar, Pitch, and Paint—Are removed from clothing by means of various liquids, such as ether, chloroform, oil of lemons, petroleum, benzine, etc. The last-named article is generally preferred, on account of its low price although the others are occasionally used for expensive fabrics. Chloroform, for instance, removes in a short time the oldest paint-stains. We think no sort of soap can replace these liquids to any advantage.

---

Boudet's Depilatory.

Crystalized sulphide of sodium 3 parts.
Quick-lime ....................10 "
Starch ........................11 "

Reduce the articles separately to fine powder, mix, and keep the mixture in a well-stoppered vial. When wanted, the powder is made into a paste with a little water, and applied to the skin; but it is important not to leave in contact for more than two to four minutes.

---

Shoe Blacking.—The following is very simple, and is said to give satisfaction.

Ivory black.................2 pounds.
Molasses...................1 pound.
Olive-oil,
Sulphuric acid, of each.......4 ounces.
Water, sufficient.

Add the acid to the ivory-black by small portions. When the effervescence has ceased, mix in thoroughly the molasses, the oil, and, lastly, enough water to bring the mixture to the desired consistence.

---

A Simple Remedy For Dandruff.—There are doubtless few persons, especially among gentlemen, who do not suffer from the inconvenience of dandruff. Physicans seem to consider it not of sufficient importance to engage their attention, and the poor victims are left either to practice their virtue of endurance, or for a cure, to try some of the many nostums advertised in the public prints.

The intolerable itching which frequently accompanies the troublesome complaint is not the only unpleasant feature, as, to persons of any pretensions to neatness, the appearance of the white scales on the coat collar and shoulders is very objectionable.

The writer, during a number of years, tried the different alcoholic solutions of castor-oil, and many other preparations, without permanent benefit, and, as a last resort, was led to adopt the plan of cleaning the scalp with borax and carb. potassa. This proved effectual, but after a persistent treatment of some months the hair became sensibly thinner, and perhaps would have soon disappeared altogether.

The belief that dandruff arises from a disease of the skin, although physicians do not seem to agree on this point, and the knowledge that the use of sulphur is frequently attended with very happy results in such diseases, induced me to try it in my own case. A preparation of one ounce of flowers of sulphur and one quart of water was made. The clear liquid was poured off, after the mixture had been repeatedly agitated during intervals of a few hours, and the head was saturated with this every morning. In a few weeks every trace of dandruff had disappeared, the hair became soft and glossy, and now, after a discontinuance of the treatment for eighteen months, there is no indication of the return of the disease. I do not pretend to explain the *modus operandi* of the treatment, for it is well known that sublimed sulphur is almost or wholly insoluble, and the liquid used was destitute of *taste*, *color*, or *smell*. The effect speaks for itself.—*Journal of Pharmacy.*

---

COLORING WASH FOR FENCES AND OUT-BUILDINGS.—The following cheap and excellent wash for wooden fences and buildings owes its durability chiefly to the *white vitriol*, which hardens and fixes the wash:—

Take a barrel and slake one bushel of freshly burned lime in it, by covering the lime with boiling water. After it is slaked, add cold water enough to bring it to the consistency of good whitewash.

Then dissolve in water, and add one pound of white vitriol (sulphate of zinc) and one quart of fine salt.

To give this wash a *cream* color, add one half a pound of yellow ochre (in powder). To give it a *fawn* color, add a pound of yellow ochre and one fourth pound of Indian red. For a handsome gray *stone* color, add one half pound French blue, and one fourth pound of Indian red. A *drab* will be made by adding one half pound of burnt sienna, and one fourth pound Venetian red.

For brick or stone, use half a bushel of lime, and half a bushel of hydraulic cement.

---

A BREAKFAST DISH.—This is from a good foreign authority: "Bruise into a saucepan 4 oz. cheese, 2 oz. butter, a pint of water, with a little salt; boil gently, adding by degrees as much flour as would thicken it; let it dry on a stove until it is like thick new butter; then add two or three eggs and a little cayenne"

TO REMOVE THE IRON TASTE FROM NEW KETTLES.—Boil a handful of hay in them, and repeat the process if necessary, Hay water is a great sweetener of tin, wooden, and iron ware. In Irish dairies everything used for milk is scalded with hay water.

---

CHICKEN CHEESE.—Boil two chickens till tender, take out all the bones and chop the meat fine, season to your taste with salt, pepper, and butter, pour in enough of the liquor they were boiled in to make it moist, put into whatever mould you wish, and when cold turn out and cut into slices.

---

A SIMPLE TRAP FOR NIGHT-FLYING INSECTS.—A writer in *Les Mondes* says, that he is enabled to materially reduce the number of insects which prey upon the flowers and fruits of his garden, by covering the inside of an old tub with liquid tar, and at twilight putting a lighted lantern within, leaving the whole out overnight. The bugs, attracted by the light, are caught and held fast by the tar.

---

PICKLING GREEN CORN.—This is a much cheaper method of preparing corn for winter use than canning it. When the corn is a little past the tenderest roasting-ear state, pull it; take off one thickness of the husk, tie the rest of the husk down at the silk end in a close and tight manner; place the ears in a clean cask or barrel compactly together, and put on brine to cover the same of about two thirds the strength of meat pickle. When ready to use in winter, soak in cold water over night, and if this does not appear sufficient, change the water and freshen still more. Corn prepared in this way is excellent, very much resembling fresh corn from the stalk.

---

TANNING LEATHER.—It is often a matter of both convenience and economy in the household and on the farm to be able to do a little tanning; so we give here an approved receipt which may prove useful. Soak the skin or hide eight or nine days in water, then put it in lime; take it out, and remove the hair by rubbing it, and soak it in clear water until the lime is entirely out: Put one pound of alum to three of salt, dissolve in a vessel sufficiently large to hold the hide; soak the hide in it three or four days, then take it out, let it get half dry, and then beat or rub it until it becomes pliable. Leather prepared by this process will not do well for shoes, but answers for hamestrings, back bands, and various other purposes on the farm.

---

COFFEE AND MILK.—Several years ago, a foreign chemist—a German, if we remember right —advanced the theory that coffee is not often injurious unless taken with milk. Of course, there are exceptional cases in which it does not agree with a person in any form, but as a rule t is its combination with milk that troubles the digestive organs. There may be some grounds for this opinion. We have known of at least two persons who were obliged to give up the use of coffee, which they had always taken with milk, and who were led to try the experiment of drinking it without milk After using the *cafe noir* for a year or more they find that it agrees with them perfectly, but a single cup of *cafe au lait* brings back the old digestive difficulties.

We refer to these cases because we see that M. Marchand, a chemist of Fecamp, has recently come to a similar conclusion concerning the mixture of coffee and milk. He does not appear to be aware that the idea is not a new one, but it is nothing strange for a Frenchman to be ignorant of scientific researches in other countries.

---

FAT MEAT FOR COMSUMPTIVES.—A taste for fat meat is unfortunately not universal among children, but when it shows itself it is often most universally repressed by parents. This taste is another expression of the wants of the living system which we cannot disregard with impunity. Without fats the organism cannot be built up in perfection. Fats counteract the tendency to consumption. Observation has established the interesting fact that persons who in early life show a taste for fat meat seldom fall victims to that disease ; and, *vice versa*, that consumptives have generally shown an early repugnance to such food. There can be no question as to the lesson taught by this fact—that when the appetite exists it ought to be indulged, and that it ought, if possible, to be created when wanting, by tonics and abundant exercise in the open air.

---

WHY A CHILD LOVES SUGAR.—The craving of children for sweets is well known to be one of the most imperious of their appetites. It has reference probably to that ceaseless activity which especially characterizes the age of childhood. It may be that sugar performs in their systems the part enacted by fatty substance in the bodies of adults. As it undergoes oxidation—is burnt up, circulating with the blood—it may be the source of the power which enables them to keep in motion from morning to night. Besides this, it is known that it renders easier and more perfect the digestion of the albuminous food upon which their growth depends. In respect to these offices it is therefore nearly essential to their well-being. And yet how strong, for generations, has been the prejudice against sugar! Under what difficulties, and in the face of what discouragements and protests, have our children obtained the luxury!

---

NEW METHOD OF MAKING BEEF-TEA.—Dr. H. C. Wood (*New Remedies*) has invented the following process for making beef-tea : Take a thin rump-steak of beef, lay it upon a board, and with a case-knife scrape it. In this way a red pulp will be obtained, which contains pretty much everything in the steak, except the fibrous tissue.

Mix this red pulp thoroughly with three times its bulk of cold water, stirring until the pulp is completely diffused. Put the whole upon a moderate fire, and allow it to come slowly to a boil, stirring all the time to prevent the caking of the pulp. As soon as it has boiled remove from the fire. Season to taste. In using this do not allow the patient to strain it, but stir the settlings thoroughly into the fluid. One to three fluid ounces of this may be given at a time.

---

TO RESTORE SCRATCHED FURNITURE.—Scrape one pound of beeswax into shavings in a pan; add half a gallon of spirits of turpentine, and one pint linseed oil. Let it remain twelve hours, then stir it well with a stick, into a liquid; while stirring, add one quarter pound of shellac varnish and one ounce alkanet root. Put this mixture into a gallon jar, and stand it before the fire, or in an oven, for a week (to keep it just warm),shake it up three or four times a day. Then strain it through a hair sieve and bottle it. Pour about a teaspoonful on a wad of baize, go lightly over the face and other parts of mahogany furniture, then rub briskly with a similar wad dry, and in three minutes it will produce a dark brilliant polish unequaled.

